# Trial management: we need a cadre of high-class triallists to deliver the answers that patients need

**DOI:** 10.1186/s13063-019-3451-z

**Published:** 2019-06-13

**Authors:** Danielle Beaumont, Monica Arribas, Lauren Frimley, Eni Balogun, Ian Roberts, Haleema Shakur-Still

**Affiliations:** Clinical Trials Unit, Department of Population Health, London School of Hygiene and Tropical Medicine, Keppel Street, London, WC1E 7HT UK

**Keywords:** Trial management, Professionalisation, Clinical trials

## Abstract

Expert trial managers with the training and experience to overcome operational challenges are often the difference between the success and failure of a clinical trial. Considerable importance is given to the beginning and the end of the clinical trial process, with those responsible for writing a protocol, obtaining funding and analysing the data all being rewarded when the results are published. Yet, trial managers are often overlooked in terms of recognition, value and status. This article highlights some of the key barriers to achieving this and makes suggestions on how they can be addressed within clinical trials units registered with the UK Clinical Research Collaboration.

## Background

Large randomised trials are used to identify modest but worthwhile treatment effects and so practical ways to increase the size of trials are needed [1]. Recruiting large numbers of participants often requires the trial to be multicentre and international, which brings a range of operational challenges, including the need to meet regulatory and ethical requirements, supply the trial materials, overcome language barriers and other challenges arising from the need to scale up recruitment. Expert trial managers with the training and experience to overcome these challenges are often the difference between success and failure.

In 2008, 48,295 studies were registered in ClinicalTrials.gov, and by August 2018, this had increased to 282,848 [2]. The number of studies supported by the National Institute of Health Research (NIHR) increased from 1787 in the year 2016 to 2070 by 2018. [3, 4], and funding for research from the Medical Research Council has increased from £233 million to £337 million in 10 years [5]. With the number of trials increasing, many more expert trial managers are needed, especially to ensure that large trials are conducted properly.

Considerable importance is given to the beginning and the end of the clinical trial process, with those responsible for writing a protocol, obtaining funding and analysing the data all being rewarded when the results are published. In the recently published WOMAN trial [6] for example, the project management plan allocated 9 months for protocol development and 6 months for preparation of the manuscript. However, what dictates a successful trial is the action taken between when the proposal is funded and when it is analysed, i.e. the conduct of the trial, which for the WOMAN trial amounted to 6.5 years. Trial managers, utilising their unique skillset and expertise, usually oversee the conduct of a trial. Yet, trial managers are usually overlooked in terms of recognition, value and status. A recent correspondence in *The Lancet* called for a cadre of high-class clinical triallists (expert trial managers) who can deliver successful trials to maximise patient benefit [7]. Recognition of the work of trial managers and continued progression and development within trial management are essential for achieving this. The aim of this paper is to highlight some of the key barriers to achieving this and to make suggestions on how they can be addressed. In particular, we will draw on our experience of working on academic trials within a clinical trials unit (CTU) registered with the UK Clinical Research Collaboration.

### The role of a trial manager in a large-scale trial

In response to the call from Peto and Baigent [1], we have conducted several successful large-scale randomised trials to answer important questions for patients. We recruited over 50,000 patients over 16 years to the CRASH-1 [8], CRASH-2 [9] and WOMAN trials [6]. All these trials recruited on time, to target and within budget and importantly, all these trials have changed clinical practice with international treatment guidelines updated to incorporate the trial results. Furthermore, this work continues with the CRASH-3 [10] and HALT-IT [11] trials, which are due to end recruitment in 2019, by which time the total number of patients recruited will be about 75,000 (Table 1).

**Table 1:** Overview of the CRASH-1, CRASH-2, WOMAN, CRASH-3 and HALT-IT trials

	CRASH-1	CRASH-2	WOMAN	CRASH-3	HALT-IT
Dates (recruitment)	1999–2004	2005–2010	2010–2016	2012–2019	2013–2019
Patients	10,008	20,211	20,000	13,000	12,000
Sites	239	274	193	175	161
Countries	49	40	21	29	14

Without skilful management, which is the domain of the expert trial manager, these trials would not be the big, multicentre, international, successful trials they are. Project management expertise alone is not enough to deliver multimillion-pound trials. A sound understanding of trial design and methodology, research operations and logistics, and the unique research context in each country taking part in the trial are also needed.

**Fig. 1:**
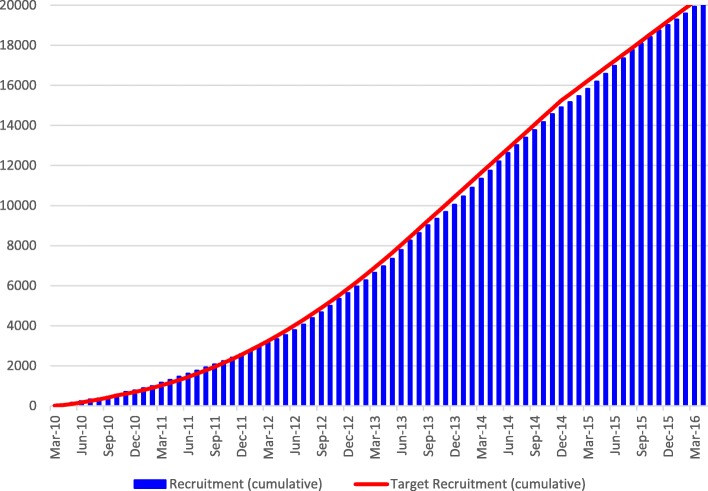
Cumulative recruitment for the WOMAN trial

**Fig. 2:**
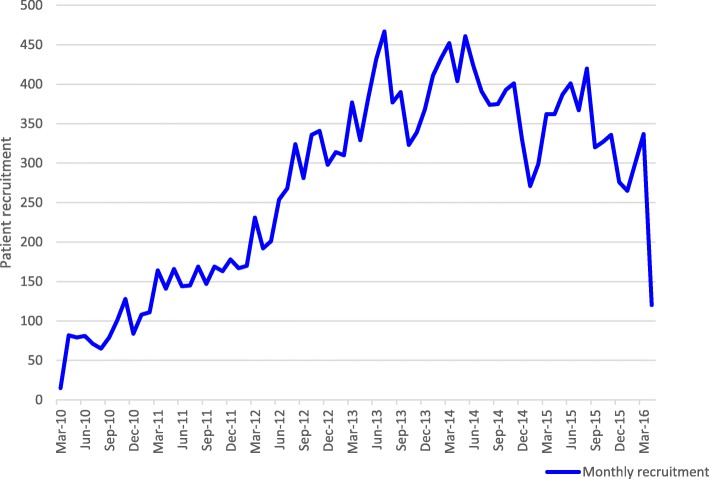
Monthly recruitment for the WOMAN trial

Patient recruitment, which is primarily the responsibility of the trial manager, is vital to the success of any trial. The NIHR Human Tissue Authority recommends that all primary research projects appoint a dedicated project/trial manager. The NIHR-funded STEPS study, which aimed to identify factors associated with good and poor recruitment to multicentre trials, showed that trials that recruited successfully had a dedicated trial manager [12]. The published manuscript of the WOMAN trial simply states that ‘between March 2010, and April 2016, 20 060 women were enrolled’. This statement gives no insight into the intricacies of this responsibility and conceals the reality of the day-to-day challenges faced by trial managers in achieving the steady increase over time that is shown in the cumulative recruitment graph for the trial (Fig. 1). Figure 2 shows the constant fluctuation in monthly recruitment into the trial during its lifetime. To keep the trial on track required persistent effort in dealing with various threats to recruitment in participating hospitals or whole countries, from political upheaval and natural disasters, to clinical trial supplies being blocked by customs authorities. Dealing with such threats whilst ensuring that the trial remained on track required critical thinking and creative solutions.

Developing and managing the patient recruitment strategy is only one part of what a trial manager must do to deliver a multicentre international trial successfully, such as the WOMAN trial. In that trial, the trial manager also had to: (1) develop trial procedures applicable to both resource-rich and resource-poor settings, (2) create, train and motivate a team (spread across the coordinating centre, national centres and sites), (3) ensure follow-ups were completed, (4) comply with all legal and ethical requirements across 21 countries, (5) arrange the manufacture and shipment of trial drugs, (6) report to all oversight committees and funders, (7) monitor trial activities, (8) analyse and interpret data on recruitment, which includes centrally monitoring recruitment statistics and the quality of the data, (9) ensure participants’ human rights are protected in line with good clinical practice guidelines and (10) ensure the budget was managed properly. It is clear a trial manager’s role is intellectual, unique, diverse and complex.

### Funders need to value the skills of trial managers

In general, trial managers are handed a trial for which the funding decisions have already been made. They are then asked to deliver a workable protocol and produce valid data ready for analysis and publication. However, funders should require that the team of academics submitting a proposal have involved a trial manager. The application should name the trial manager and describe their specific expertise, experience and contribution to the proposal. This will ensure that the best application is submitted with appropriate input from all experts, for example, those with clinical, statistical and trial management skills.

Once a proposal is submitted, funders rarely involve expert trial managers at the board review stage. After an online search of their websites on 30 May 2018, we reviewed the composition of the funding boards of three main UK funders: the Medical Research Council, NIHR and the Wellcome Trust. Across the three funding boards, we identified 77 committees, panels and expert review groups with 1134 members listed. Some members sat on more than one board. Our search showed that none of the funding boards had a member with a job title of ‘trial manager’ or ‘trial management expert’. Funding board members are usually selected for their expertise, experience and specialist skills. Trial managers with their unique experience in conducting trials are generally excluded from this early process.

Another key aspect in which funders can have an impact is investing in the development of the body of knowledge needed to manage trials, which could prevent many trials from failing. The call for more evidence to support decisions on the design, conduct and reporting of clinical trials has been ongoing for over 30 years [13, 14]. The recent Trial Forge initiative will develop the science of trial methodology. It aims to provide the evidence needed to improve trial efficacy and for conducting studies within a trial (SWATs). It is a small start in developing methodology closely aligned to a trial manager’s skillset [15]. This initiative is evaluating and exploring alternative ways of delivering and organising trials. It recommends that SWATs should be embedded in all funded trials. Moreover, a recent study highlighted concerns from CTUs regarding applications for SWATs. Examples included the main funding application being rejected due to the additional costs of a SWAT and insufficient time being allocated to develop the SWAT due to tight submission timelines [16]. Funders commented that there is a misconception surrounding what money is available for. However, funders should realise that the SWAT component of a trial can be vital and they should fund SWATs adequately. They should ensure there are sufficient resources to cover this additional important work. Funders could also allow for a SWAT to be embedded later or actively request that a SWAT is included in funding applications.

### The role of journals in recognising the importance of trial management

The structure of a traditional scientific manuscript favours the scientific and statistical aspects. Neither successful nor problematic trial management aspects are expected to be reported in a clinical trial publication. Some trials are delivered successfully but many more encounter difficulties in recruitment, consent, adherence to the intervention, follow-ups, regulatory and ethical issues, and quality management. The opportunity to learn from clinical trials is enormous. The inclusion of trial management methodology as part of a clinical trial publication will ensure that the knowledge acquired during the conduct of each trial is not lost and can be applied in future trials and subsequently used to develop trial methodology further. Editors of medical journals need to consider the importance of how good research is actually carried out and should require that trial management methods are included in descriptions of trials in high-quality journals. Whilst more papers are describing trial management [17–19] as good practice, doing so is not mandatory. Transparency in the publication of trial management methods is not expected, unlike the publication of basic research.

Authorship is important in recognising and attributing contribution in a trial manuscript. During the peer review, reviewers and journal editors should ensure that there is an appropriate acknowledgement of the trial manager and require that the trial manager is a named author. In addition, a line acknowledging the trial management team should be permitted.

### Gender inequality and the role of the trial manager

In a clinical trial, the chief investigator is most likely to be a he as women remain underrepresented in leadership roles [20, 21] and as funding recipients [22, 23]. In contrast, trial managers are ‘she, seldom he’ [24]. On 30 May 2018, we reviewed the websites of 46 CTUs registered with the UK Clinical Research Collaboration. Staff lists were available for 37 of them. Based on title, name and photographs, we identified that approximately 83% of trial managers were female. This leads to a hierarchical structure and a power imbalance between trial managers and chief investigators. Because the predominantly female role of trial management is situated within a stark sexual division of labour alongside the predominantly male role of chief investigator, gender may account for a significant part of the inequalities. Whilst we acknowledge gender imbalance and inequalities are not specific to trial managers but widespread across different industries, to ensure continued progression and development of talented trial managers, both male and female, and to develop the leaders needed, it is important to address the reasons for this. For example, is the gender imbalance of trial managers attributed to the perceived skillset for the role? What can be done to ensure that there are more females in positions of leadership within trial management? Women remain underrepresented as recognition award recipients [25–28].

It is important to examine this gender imbalance and to determine the impact that such an imbalance might have on the continued progression and development of trial managers. For instance, is there less focus on creating pathways that would facilitate interested trial managers to progress to chief investigator roles within CTUs because of a general perception that women are stronger and more useful in support roles?

Trial managers themselves might hold some responsibility for the lack of recognition of their contribution in clinical trials. It is known that women talk down their achievements and undervalue themselves when working in a successful group alongside men [29].

Trials are delivered through collaborative working. A collaboration is an interdependence that requires the complementarity of roles [30]. The notion that the chief investigator’s role and the trial manager’s role complement each other is essential in delivering clinical trials successfully.

### Career structure and professional recognition for trial managers

Trial managers are predominantly accidental triallists, having learnt their skills from their peers through on the job training supported with in-house training. However, trial managers at every level should develop the skills needed to deliver high-quality clinical trials confidently and competently. It is crucial that training opportunities and continued professional development are available and experiences are shared to develop the expert trial managers of the future.

The NIHR Task, Knowledge and Competency Framework for trial managers identifies three competency levels: novice, experienced and senior [31]. Trial managers working within CTUs are provided with support and a structure around these roles to achieve progression. Yet, whilst the framework is a useful tool to progress individuals through from early to perhaps mid-career, as it currently stands, expert trial managers hit a glass ceiling. The framework does not outline further opportunities for progression in which CTUs can offer continued support, nor does it recognise that trial managers can become leaders in their field.

Appropriate career structures are needed for trial managers to develop into leaders, achieve career success and build the knowledge base to support the profession. One approach used by the London School of Hygiene & Tropical Medicine’s CTU is to develop the expertise of trial managers through an academic pathway, which allows trial managers to complete appropriate postgraduate education and to develop the methodological skills needed to build the body of knowledge and evidence base for efficient and expert trial management. However, it is recognised that not all trial managers want an academic career and alternative pathways are also needed with a focus on the management and leadership skills required to deliver a trial successfully. Practical support should also be provided within institutions for expert trial managers to develop their own funding proposals for their own research (for instance, developing SWATs), which would be aimed at developing the overall body of knowledge and allowing them to develop as leaders.

The last few years have seen some growth in training and educational opportunities for trial managers. The UK Trial Managers Network has developed workshops to support trial managers’ core work [32]. Several MSc programmes covering clinical trials [33–36] and short courses on clinical trial management [37–40] are now available. However, education programmes and training can be expensive. Research funders and employing institutions need to invest in the training of trial managers to ensure the successful delivery of clinical trials. NIHR leadership programmes are available for early career researchers [41]. Such leadership programmes are also needed to develop expert trial managers.

We propose that one way to formally address training, education and career structure needs and to recognise the expertise of trial managers is professional accreditation of trial managers. Steps have already been made by the UK Trial Managers Network to develop a professional accreditation scheme for trial managers. The network is attempting to formalise this process and its continuing work must be appropriately supported. Trial managers need a formal body to represent their work, which the UK Trial Managers Network has provided to date. However, what is needed to support professionalisation is a body that also maintains oversight of the knowledge, skills, conduct and practice of trial management.

## Conclusion

Trial managers are vital to the success of clinical trials. The contribution of trial managers needs to be recognised, rewarded and valued by funders, journals, academic institutions and their peers. This will signal their status, their confidence, the trust placed in them and their professional autonomy. Academic institutions hosting CTUs need to provide training and development opportunities that will facilitate progression beyond trial management to chief investigator or a relevant academic role if desired. There should be appropriate career pathways for trial managers wanting to remain within the field as experts. The professionalisation of trial management should be enhanced through education and training. These measures will go some way in developing a cadre of high-class triallists who will be able to ensure the success of future clinical trials that deliver the answers that patients need.

## Data Availability

Not applicable

## Abbreviations

CTU: Clinical trials unit
NIHR: National Institute of Health Research
SWAT: Study within a trial
